# Inhibition of Nucleotide Biosynthesis Potentiates the Antifungal Activity of Amphotericin B

**DOI:** 10.1371/journal.pone.0087246

**Published:** 2014-01-30

**Authors:** Dithi Banerjee, Lauren Burkard, John C. Panepinto

**Affiliations:** Department of Microbiology and Immunology, Witebsky Center for Microbial Pathogenesis and Immunology, University at Buffalo, The State University of New York, Buffalo, New York, United States of America; Louisiana State University, United States of America

## Abstract

The polyene antifungal agent Amphotericin B exhibits potent and broad spectrum fungicidal activity. However, high nephrotoxicity can hinder its administration in resource poor settings. Quantification of early fungicidal activity in studies of HIV patients with cryptococcosis demonstrate that 5-Fluorocytosine therapy in combination with Amphotericin B results in faster clearance than with Amphotericin B alone. *In vitro* synergy between the two drugs has also been reported but the mechanism by which 5-Fluorocytosine synergizes with Amphotericin B has not been delineated. In this study we set out to investigate the effect of genetic mutation or pharmacologic repression of *de novo* pyrimidine and purine biosynthesis pathways on the Amphotericin B susceptibility of *Cryptococcus neoformans.* We demonstrate that a *ura-* derivative of wild type *Cryptococcus neoformans* strain H99 is hypersensitive to Amphotericin B. This sensitivity is remediated by re-introduction of a wild type *URA5* gene, but not by addition of exogenous uracil to supplement the auxotrophy. Repression of guanine biosynthesis by treatment with the inosine monophosphate dehydrogenase inhibitor, mycophenolic acid, was synergistic with Amphotericin B as determined by checkerboard analysis. As in *Cryptococcus neoformans*, a *ura* ^−^ derivative of *Candida albicans* was also hypersensitive to Amphotericin B, and treatment of *Candida albicans* with mycophenolic acid was likewise synergistic with Amphotericin B. In contrast, neither mycophenolic acid nor 5-FC had an effect on the Amphotericin B susceptibility of *Aspergillus fumigatus.* These studies suggest that pharmacological targeting of nucleotide biosynthesis pathways has potential to lower the effective dose of Amphotericin B for both *C. neoformans* and *C. albicans.* Given the requirement of nucleotide and nucleotide sugars for growth and pathogenesis of *Cryptococcus neoformans*, disrupting nucleotide metabolic pathways might thus be an effective mechanism for the development of novel antifungal drugs.

## Introduction

The pathogenic fungus *Cryptococcus neoformans* causes fatal meningitis in patients with defects in T cell function. Patients with HIV make up the largest population with susceptibility to cryptococcosis, and more than 600,000 patients succumb to cryptococcosis yearly [1].

The gold standard of antifungal therapy is Amphotericin B (AmB), a polyene antifungal with potent fungicidal activity against a broad range of pathogenic fungi [2], [3]. The benefit of AmB therapy can be overshadowed by its high toxicity [4], [5], with treatment requiring monitoring of patient renal function. Because the majority of cryptococcosis cases occur in resource-poor settings, treatment with AmB is often precluded due to lack of infrastructure necessary to monitor patient electrolytes.

Nucleotide biosynthesis pathways are common therapeutic targets for cancer therapy, antiviral therapy, and anti-parasite therapy [6]–[13]. A single nucleoside analogue, 5-fluorocytosine (5-FC) has been used as anti-fungal therapy [14]. The basis of 5-FC antifungal activity is its incorporation into cellular nucleotide pools through the pyrimidine salvage pathway after bioconversion to the active metabolite, 5-fluorouracil. High rates of spontaneous resistance have hampered the use of 5-FC as monotherapy for fungal infections [14], [15]. Combination therapy of AmB with 5-FC increases the early fungicidal activity of AmB in HIV patients with cryptococcosis [16], [17], and synergy between these two antifungal drugs has been demonstrated *in vitro* [18]–[21]. The mechanism underlying this synergy has not been described.

*Cryptococcus neoformans* encodes the necessary enzymes for *de novo* synthesis and salvage of both purine and pyrimidine nucleotides. Mutation of *de novo* pyrimidine synthesis has been demonstrated to reduce virulence in mouse models of cryptococcosis [22], suggesting that pyrimidine salvage was insufficient to support full virulence of *C. neoformans.* Likewise, *de novo* synthesis of guanosine was also required for full virulence [23]. In this study, we investigated the impact of perturbations in *de novo* synthesis of both purine and pyrimidine nucleotides on the anti-cryptococcal activity of AmB. Loss of pyrimidine biosynthesis through mutation of the *URA5* gene resulted in increased susceptibility of *C. neoformans* to AmB that was reversed by re-introduction of a wild type *URA5* gene. Supplementation of pyrimidine auxotrophy through the addition of uracil or uridine to the medium was not able to reverse the hypersensitivity to AmB, suggesting that an intact salvage pathway is insufficient to support the pyrimidine draw required for wild type resistance to AmB. Likewise, inhibition of *de novo* synthesis of guanosine nucleotides by treatment with mycophenolic acid (MPA) also resulted in increased susceptibility to AmB, with the compounds displaying synergistic interactions in checkerboard MIC assays. A *ura* ^−^ mutant of *Candida albicans* was likewise hypersensitive to AmB, but uracil as well as uridine supplementation was able to reverse this effect. Treatment with MPA and 5-FC also reduced AmB MIC for *C. albicans*, whereas neither drug affected *Aspergillus fumigatus* AmB susceptibility. Additionally, the *C. neoformans ura* ^−^ mutant was found to have capsular defects and supplementation with uracil or uridine restored capsule formation suggesting that capsule biosynthesis draws from the pool of salvaged pyrimidine nucleotides.

## Materials and Methods

### Strains

Studies with *Cryptococcus neoformans* were performed with wild type *Cryptococcus neoformans* var *grubii* strain H99. A *C. neoformans ura* ^−^ mutant strain was generated by screening for spontaneous *ura* ^−^ mutant on Yeast Extract Peptone Dextrose (YPD) agar supplemented with 1 mg/mL 5-Fluoroorotic acid. To confirm a typical mutation of the *URA5* gene, PCR primers (5′-ATTGGAGTTCGCTTACCTTTGG-3′, 5′-ACTTAAGTTCCTTTGATACAAGC-3′) were designed to amplify the *URA5* gene and the purified PCR product was transformed in *ura* ^−^ mutant by electroporation [24] following which complement strains were screened for the ability to grow in minimal media, in the absence of exogenous uracil, confirming uracil prototrophy.

Wild type *Candida albicans* SC5314 and *C. albicans ura* ^−^ mutant CAI-4 were kind gifts from Dr. Mira Edgerton, University at Buffalo [25]. Wild type *Aspergillus fumigatus* strain H237 used in this study was a gift of Dr. Judith Rhodes, University of Cincinnati. All yeast strains were stored in YPD-30% glycerol at −80°C and were subcultured on YPD agar prior to the experiments. The *A. fumigatus* strain was stored at 4°C on potato dextrose agar slants, subcultured on same media and incubated for 4–7 days for optimal conidiation prior to experiments. All assays were performed in triplicate.

### Media and Chemicals

Yeast strains were sub-cultured on Yeast Extract Peptone Dextrose (YPD) agar (Difco, MD, USA) and *A. fumigatus* strain was grown on Potato Dextrose Agar (Sigma Aldrich). YPD broth was used for growing starter cultures of all yeast strains. Antibiotic medium 3 (AM3) (Difco, MD, USA) buffered to pH 7.0 with 10 mM phosphate was used for E-test with all strains. For checkerboard assays with combination of AmB and MPA, AM3 broth supplemented with 2% dextrose (AM3-2% Dex) was used. RPMI 1640 medium (Gibco, Grand Island, NY) supplemented with 2% dextrose (RPMI1640-2% Dex) and buffered by 0.165 3-(N- morpholino)propanesulfonic acid (MOPS) and 10 M NaOH to pH 7.0 was used for analysis of AmB and 5-FC combination checkerboard assays.

E-test strips containing graded concentration (0.002 µg/mL to 32 µg/mL) of AmB were supplied by (Biomerieux). AmB was obtained from Cellgro, Mediatech Inc. as an aqueous solution (250 µg/mL). Stock solution of 100 µg/mL was prepared in sterile distilled water; filter sterilized and stored at −20°C. MPA (25 mg/mL) (MP Biomedicals, LLC) was dissolved in 99% ethanol to make a 12.5 mg/mL stock solution, 100 µL aliquots of the same was placed in microcentrifuge tubes and stored at −20°C. 5-FC was purchased from Sigma Aldrich and dissolved in water to prepare a 2 mg/mL stock solution. Guanine, uracil and uridine bases (Sigma Aldrich) were obtained as reagent grade powders. 20 mg/mL guanine stock solution was made by reconstituting in 0.015 mM KOH and 20 mM uracil and uridine stock solution was prepared in water and stored at room temperature.

### Susceptibility Testing

1. E-test. In vitro antifungal susceptibility to AmB alone or in combination with MPA was determined by E-test according to the manufacturer’s protocol. For *C. neoformans* and *Candida albicans* isolates, cells were grown to mid-log phase in YPD broth, washed and diluted to an OD_600_ = 0.5. *A. fumigatus* conidia were dissolved in sterile distilled water, filtered through sterile Miracloth and enumerated in hemocytometer slide to yield a final inoculum size of 5×10^5^ conidia/mL. Cells were plated with sterile cotton swab to produce confluent growth on AM3 medium supplemented with or without 3.25 µg/mL MPA. Additionally, E-test with wild type *C. neoformans* was performed on AM3 medium supplemented with 3.25 µg/mL MPA and 20 µg/mL guanine. E-tests with *C. neoformans* and *C. albicans ura* ^−^ mutants were also performed on AM3 medium supplemented with 20 µM uracil and 20 µM uridine. Once plates were dry, E-test strips were placed on them and the plates were then incubated at 37°C for 48–72 hours. The MIC was determined as the lowest concentration of the drug at which the border of the elliptical zone of growth inhibition intercepted the scale on the strip [26], including any microcolonies that were present.
2. Checkerboard assay. Susceptibility to AmB and MPA were assessed by a checkerboard assay based on standard protocols proposed by CLSI for broth microdilution antifungal susceptibility test. The media used for analyzing a combination of AmB and MPA against all wild type fungal isolates was AM3-2% Dex, whereas RPMI 1640-2% Dex was used for testing combination of AmB and 5-FC against wild type *C. albicans* and *A. fumigatus*. Working solutions of each drug was prepared in the test media at a concentration of four times the targeted final concentration in the assay range. 100 µL of drug mixture with 50 µL of increasing concentrations of one drug and 50 µL of fixed concentration of the other drug used in the combination were mixed and dispensed in the 96 well microtiter plates. One row and column had 50 µL of single drug solution of each concentration. Two wells containing 100 µL each of assay medium and inoculum served as growth control (GC). A single well containing 200µL of only the assay medium served as sterility control (SC). Yeast inocula were prepared to yield a final cell concentration of 1×10^5^ CFU/mL. Final concentration of *A. fumigatus* conidia used in checkerboard assay was 1×10^5^ conidia/mL. 100 µL of the inoculum was dispensed in each well (except SC), plates were incubated in air at 37°C and read after 72 hours. Readings were performed spectrophotometrically using an automated plate reader (Spectramax M5, Molecular Devices, CA) set at 540 nm. MIC endpoint for AmB was defined as the lowest drug concentration, alone and in combination that prevented any discernible growth. For MPA and 5-FC, the MIC was defined as the lowest concentration of drug tested alone and in combination, at which the turbidity in the well was 50–80% lesser than in the control well (GC).

MIC and Fractional Inhibitory Concentration (FIC) index of each drug combination was calculated to assess drug interaction in each isolate. FIC index is the sum of FIC of each drug, which in turn is defined as the MIC of each drug when used in combination divided by the MIC of the drug when it is used alone [26], [27].

### Killing Assay

An overnight culture of wild type *C. neoformans* grown in YPD at 30°C was harvested, washed in sterile distilled water and adjusted spectrophotometrically to an OD_600_ = 1.0. An appropriate volume of the cell suspension was diluted with 120 mL of AM3 to obtain an inoculum of 1×10^5^ CFU/mL. The cell suspensions were divided equally into 12 conical tubes with one serving as control without any drugs. 30 µg/mL MPA was added alone and in combination with graded concentrations of AmB (0.3, 0.6, 0.12, 0.25 and 0.50 µg/mL) to prepare the test solutions. Growth controls and test solutions were then incubated in a shaker at 37°C. Samples were withdrawn after 6 and 24 hours of drug exposure, serially diluted and 200 µL of the suspension was plated onto duplicate sets of YPD agar. The plates were incubated at 30°C for 48 hours and colony counts were determined.

### Capsule Detection Assay

Wild type, *ura* ^−^ mutant and complement strains were grown in YPD broth till mid-log phase. India ink preparations of the three strains prior to capsule induction were done by mixing 5 µL of cells with 3 µL of stain on a slide and observed under Differential Interference Contrast (DIC). Cells were then harvested, washed twice, resuspended in sterile distilled water and adjusted to an OD_600_ = 1.0 200 µL of this cell suspension was inoculated for capsule induction in 5 mL 1X RPMI [28] or 1∶10 diluted YPD, media similar to Sabauraud Dextrose medium used for the same purpose [29]. Medium was either inoculated alone, or in combination with uracil and uridine, in a tissue culture plate or incubated at 37°C, in 5% CO_2_ for 48–72 hours. India ink preparations were made as mentioned earlier and size of capsule was reported by measuring the radius of the dye excluding region between the cell wall and the India ink dye front. Measurements were performed using LAS-AF software (Leica).

### Survival Assay

*ura* ^−^ mutant cells from capsule detection assay (1X RPMI +/− uracil and uridine in 5% CO_2_ and 37°C) were diluted 100 times and 100 µL of this suspension was plated out on YPD agar plates following 0, 24 and 48 hours of capsule induction. Plates were incubated for 48 hours at 30°C following which CFUs were enumerated.

#### Statistical analysis

For killing assays and capsule measurements, a one way ANOVA was used to assess significance. Pairwise analyses were conducted using Tukey’s t-test post-hoc.

## Results and Discussion

### Inhibition of Pyrimidine de novo Synthesis Increases Susceptibility of *C. neoformans* to AmB

**Figure 1:**
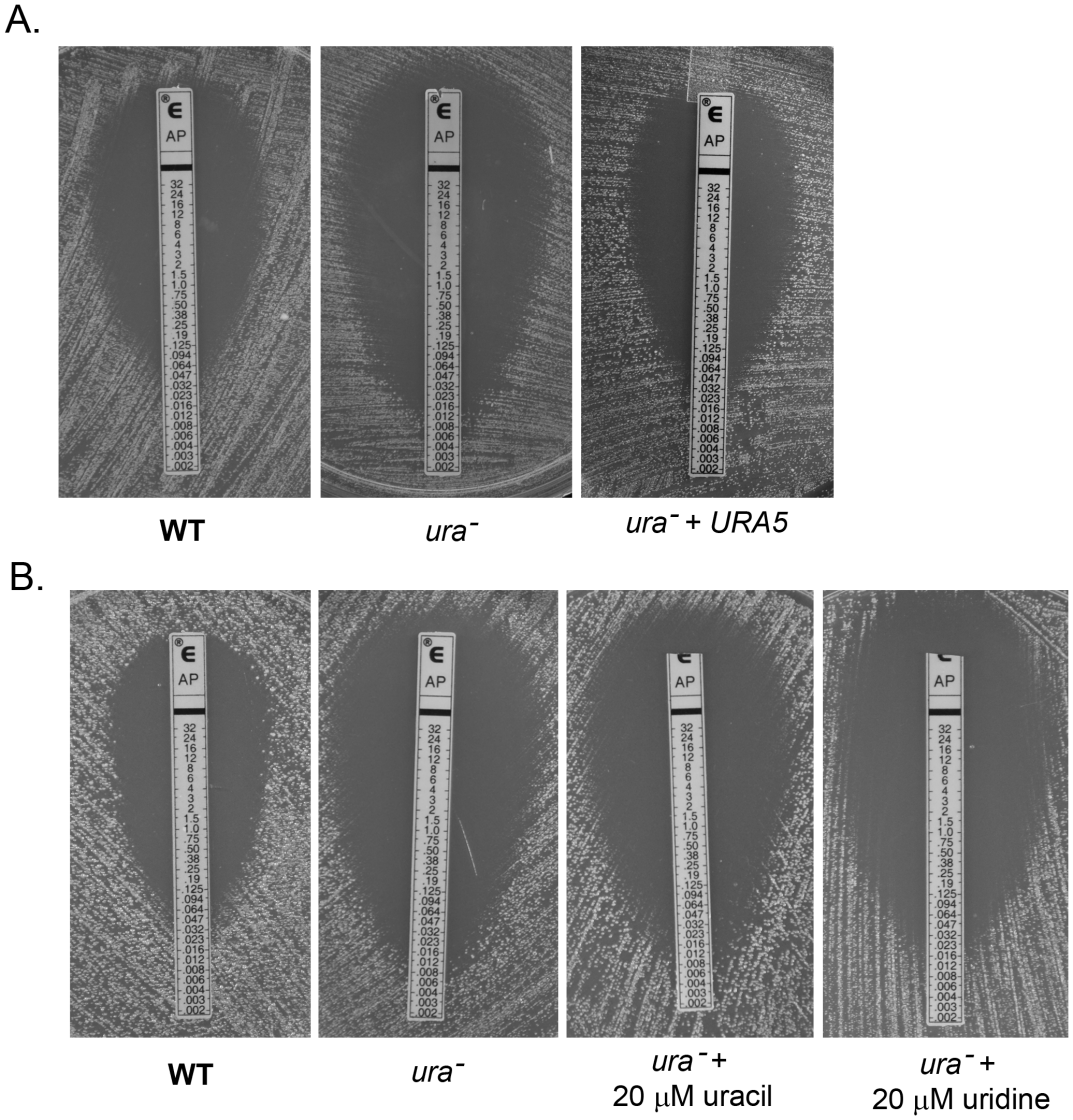
A *C. neoformans ura* ^−^ strain is hypersensitive to AmB. (A) E-Test for AmB susceptibility of *C. neoformans* wt, *ura* ^−^ mutant and *ura* ^−^ strain complemented with the wild type *URA5* gene on AM3 medium (B) E-Test for AmB susceptibility of *C. neoformans* wt and *ura* ^−^ mutant on AM3 medium supplemented with 20 µM uracil and 20µM uridine.

The combination of AmB and 5-FC exhibited synergy *in vitro* and enhanced early fungicidal activity in human studies against *C. neoformans* [16], [18], [19], [21]. To determine if this effect was specific to 5-FC, or if general perturbation of pyrimidine biosynthesis could affect AmB susceptibility, we tested the AmB susceptibility of a *ura* ^−^ mutant (pyrimidine synthesis mutant) of *C. neoformans* by E-test. As demonstrated in Figure 1-A, comparison by E-test revealed a larger zone of inhibition in the *ura* ^−^ strain as compared to wild type and complemented strain that correlated to a significant decrease in the MIC of *ura* ^−^ mutant. This was reverted in the complemented strain with an MIC value comparable to that of wild type. We know that in the absence of *de novo* synthesis, cells utilize preformed bases (either internal cellular recycled products or exogenous precursors) to synthesize nucleotides by salvage pathway [30]. We thus wanted to evaluate whether *ura* ^−^ strain (*de novo* synthesis mutant) was able to utilize salvage pathway to replenish the nucleotide pool and overcome the increased sensitivity to AmB. The addition of 20 µM uracil and 20 µM uridine (Figure 1-B) to the medium was unable to restore wild type AmB sensitivity to *ura* ^−^ mutant, suggesting that pyrimidine nucleotide salvage was not sufficient to reverse the increased susceptibility of *C. neoformans* to AmB when *de novo* synthesis of pyrimidine was absent.

During *de novo* synthesis of pyrimidine nucleotides, UMP is synthesized first which in turn becomes the precursor for UDP, UTP and CTP biosynthesis [30]. Thus, when *de novo* synthesis is perturbed, there is a reduction in the entire pyrimidine nucleotide pool which is replenished with the restoration of the pathway in the *URA5* complement. In contrast, the salvage pathway utilizes pyrimidine bases cytosine, cytidine and uridine for the synthesis of their respective nucleotides. Thus, the supplementation of *ura* ^−^ mutant with uracil alone would mainly result in the replenishment of UMP, UDP and UTP pools, whereas reduced cytidine nucleotide pools may persist. A unique feature of *C. neoformans* is its large polysaccharide capsule. UDP sugars like UDP-galactose, UDP-xylose and UDP-glucuronic acid are the activated donors and precursors of capsular polysaccharide whereas UDP - glucose and UDP-*N-*Acetylglucosamine serve the same purpose in cell wall biosynthesis [31]. Deletion of *UGD1* and *USX1* genes that encode for *C. neoformans* UDP-glucose dehydrogenase [32] and UDP-xylose synthase [33] respectively leads to acapsular, temperature sensitive and avirulent phenotypes. *URA5* mutants are also less virulent in mice [22]. Given the importance of uridine nucleotides in cryptococcal structural development, virulence and pathogenesis, it is likely that the influx of uracil in the *ura* ^−^ mutant is prioritized toward the synthesis of UMP and UDP leaving the pyrimidine pools imbalanced. This reliance on pyrimidine pools for pathogenesis reinforces the potential of pyrimidine biosynthesis as a drug target in *C. neoformans.*

To assure the effect of AmB on *ura* ^−^ mutant was not due to a global defect in growth, Fluconazole E-test was performed. No difference in fluconazole susceptibility was seen between the wild type and the *ura* ^−^ mutant (data not shown).

### Inhibition of Guanosine de novo Synthesis Renders *C. neoformans* more Susceptible to the Action of AmB

**Figure 2:**
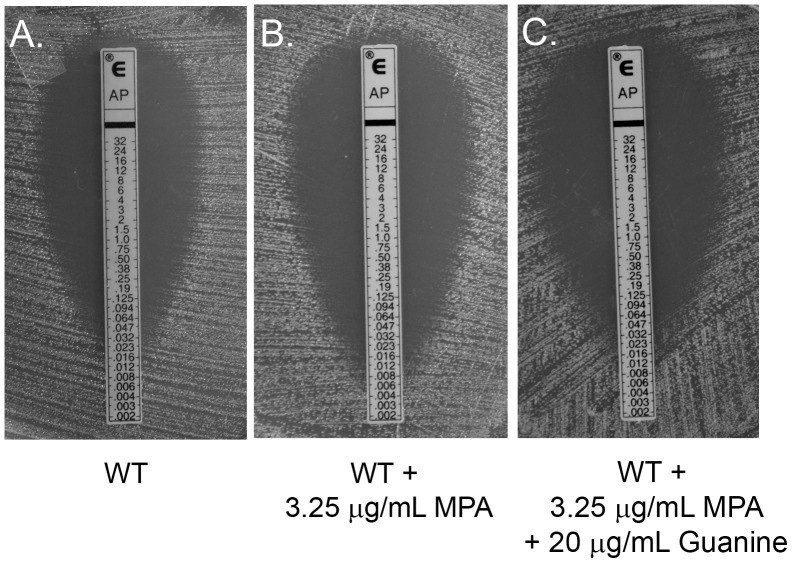
Inhibition of guanosine biosynthesis sensitizes *C. neoformans* to AmB. (A) E-Test of *C. neoformans* wt on AM3 medium (B) AM3 medium supplemented with 3.25 µg/mL MPA and (C) E-test analysis for AmB susceptibility of *C. neoformans* wt on AM3 supplemented with 3.25 µg/mL MPA and 20 µg/mL exogenous guanine.

Because inhibition of pyrimidine *de novo* synthesis increased the anticryptococcal efficacy of AmB, we next wanted to investigate whether perturbation in the purine *de novo* synthesis resulted in a similar phenomenon. To evaluate this, we performed E-test with wild type *C. neoformans* using a combination of AmB and MPA, a drug that represses *de novo* synthesis of guanosine nucleotides through inhibition of the enzyme inosine monophosphate dehydrogenase (IMPDH). We observed a larger zone of inhibition and a subsequent three-fold decrease in AmB MIC which suggested that presence of MPA potentiated AmB fungicidal activity (Figure 2-A and 2-B). Supplementation of exogenous guanine in the media reversed the effect of MPA (Figure 2-C), indicating that salvage pathway of purine biosynthesis was more functional than salvage of pyrimidines in *C. neoformans*. Addition of MPA to the growth medium had no effect on fluconazole sensitivity of wild type *C. neoformans* (data not shown), suggesting that this effect was specific to AmB. This difference between Fluconazole and AmB susceptibility is striking since both the antifungals act on ergosterol, a component of the fungal cell membrane, with the former drug inhibiting its biosynthesis and AmB binding to the membrane and resulting in loss of function. Interestingly, AmB has been reported to induce apoptosis in *C. albicans* [34] which is a novel mechanism of action for this drug. Nucleotide deficiency in the MPA treated or *ura* ^−^ mutant cells may predispose them to AmB mediated apoptosis. Future studies will focus on the mechanism by which nucleotide pools impact AmB susceptibility.

**Figure 3:**
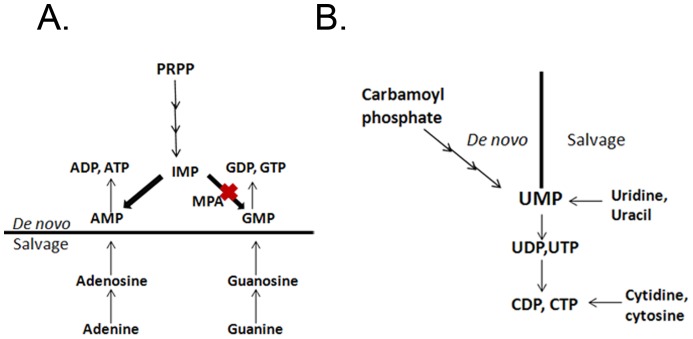
Schematic diagram of *de novo* and salvage pathways of purine and pyrimidine nucleotide biosynthesis.

Perturbation of *de novo* GTP synthesis by the loss of *IMPDH* has shown to result in defective capsule production, melanin synthesis and decreased virulence in *C. neoformans* [23]. Treatment with MPA which perturbs the same step in the pathway has demonstrated similar phenotypic effects which were recovered by the addition of exogenous guanine [23]. The ability of guanine to suppress the effect of MPA can be attributed to the general difference in the salvage pathways of purine and pyrimidine nucleotide synthesis. We know from Figure 3 that UMP is the central molecule in both *de novo* and salvage pathways from which the other uridine and cytidine nucleotides are formed in a sequential manner. Thus, in case of *ura* ^−^ mutant, there is a deficit of pyrimidine pools in general. In contrast, *de novo* synthesis and purine salvage of adenosine and guanosine nucleotides are independent of each other. As we see in Figure 3, when *de novo* synthesis of GMP is inhibited by MPA, the levels of AMP biosynthesis arm are unimpeded. There may be increased flux towards AMP synthesis due to the channeling of precursors from the upstream shared *de novo* biosynthesis pathway. When exogenous guanine is supplemented in the presence of MPA, guanosine nucleotides are salvaged specifically thereby restoring the total purine nucleotide pools and reverting the phenotype of enhanced AmB susceptibility.

**Figure 4:**
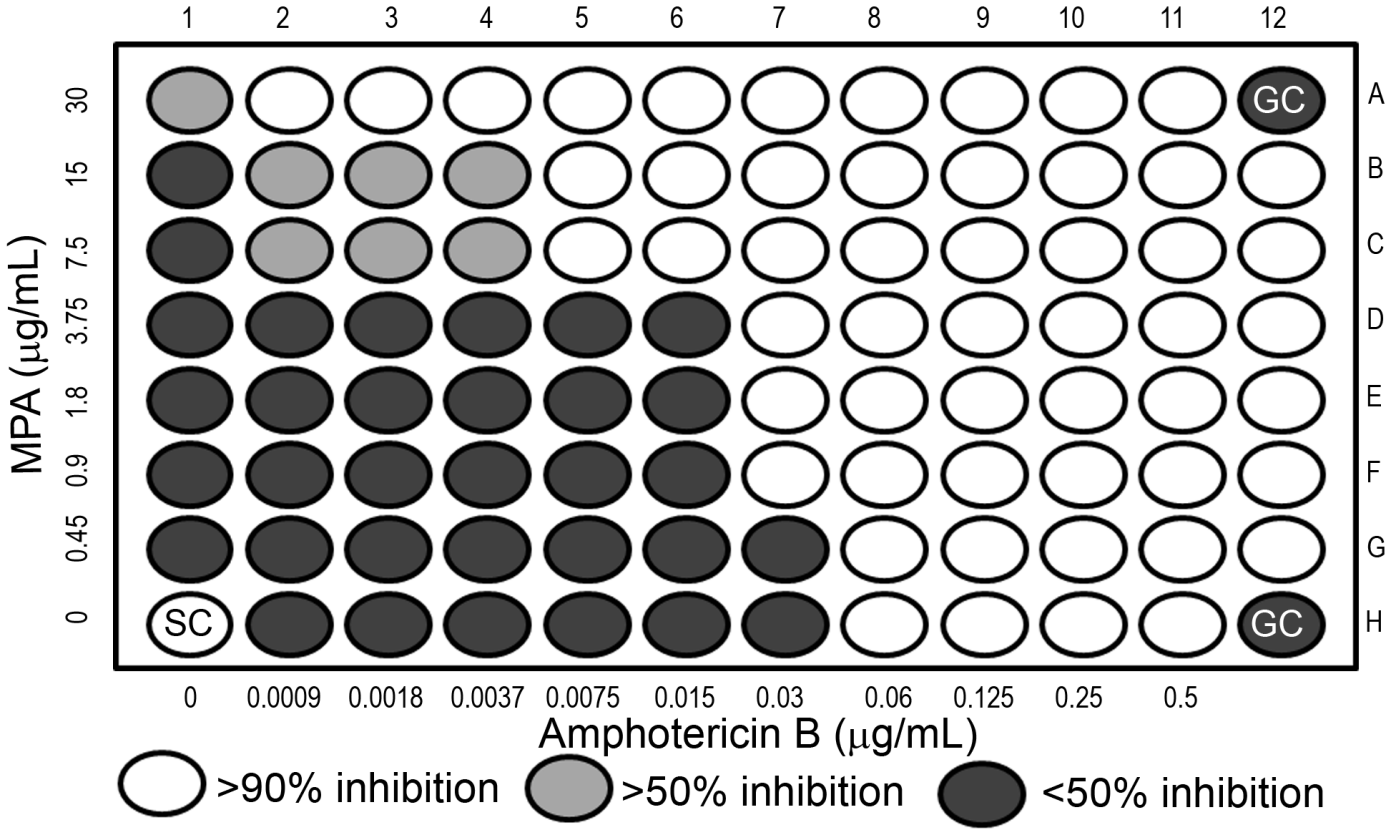
AmB and MPA synergize against *C. neoformans*. Assay range of concentrations for AmB is 0.0009 µg/mL to 0.5 µg/mL and for MPA is 0.45 µg/mL to 30 µg/mL. Well A12 is the sterility control (SC) and wells H1 and H12 are growth controls (GC) for MPA and AmB respectively.

Once the MIC of AmB was seen to be lowered with the simultaneous use of MPA, we further wanted to determine whether interactions between the two drugs were synergistic or additive. Antifungal susceptibility testing was performed with wild type by checkerboard assay with a combination of AmB (concentration ranging from.0009 µg/mL to 0.5 µg/mL) and MPA (0.45 µg/mL to 30 µg/mL). In Figure 4, the mean MIC of AmB alone from 3 independent experiments was 0.06 µg/mL while that of MPA alone was 30 µg/mL. Mean MIC was significantly lowered by approximately three-fold to.0075 µg/mL for AmB and 7.5 µg/mL for MPA when the drugs were used in combination. The FIC values from 3 replicate experiments used to classify the drug interactions are reported in Table 1. An FIC value ≤0.5 indicated synergy, >0.5 and <1.0 suggested additive interaction whereas any value between 1.0 and 4 indicated indifference and >4 indicated antagonism between the two drugs [26], [27]. The FIC index from three independent experiments were 0.375, 0.25 and 0.5 which indicated a synergistic interaction (≤0.5 is synergy) between the two drugs.

**Table 1:** Drug interaction between AmB and MPA in *C. neoformans.*

Drug (µg/mL)	Replicate 1	Replicate 2	Replicate 3
**AmB alone (MIC_A_)**	0.06	0.12	0.06
**AmB in combination (MIC_A’_)**	0.0075	0.0075	0.015
**MPA alone (MIC_B_)**	30	30	30
**MPA in combination (MIC_B’_)**	7.5	7.5	7.5
**FIC index = MIC_A’_/MIC_A_+MIC_B’_/MIC_B_**	0.375	0.25	0.5
**Interaction**	Synergy (≤5)	Synergy (≤5)	Synergy (≤5)

AmB: Amphotericin B, MPA: Mycophenolic acid.

MICs of AmB and MPA alone and in combination against *C. neoformans* in 3 separate experiments and calculated FIC values.

### Time Kill Assay shows Decreased CFU on Treatment with a Combination of Amphotericin B and MPA

**Figure 5:**
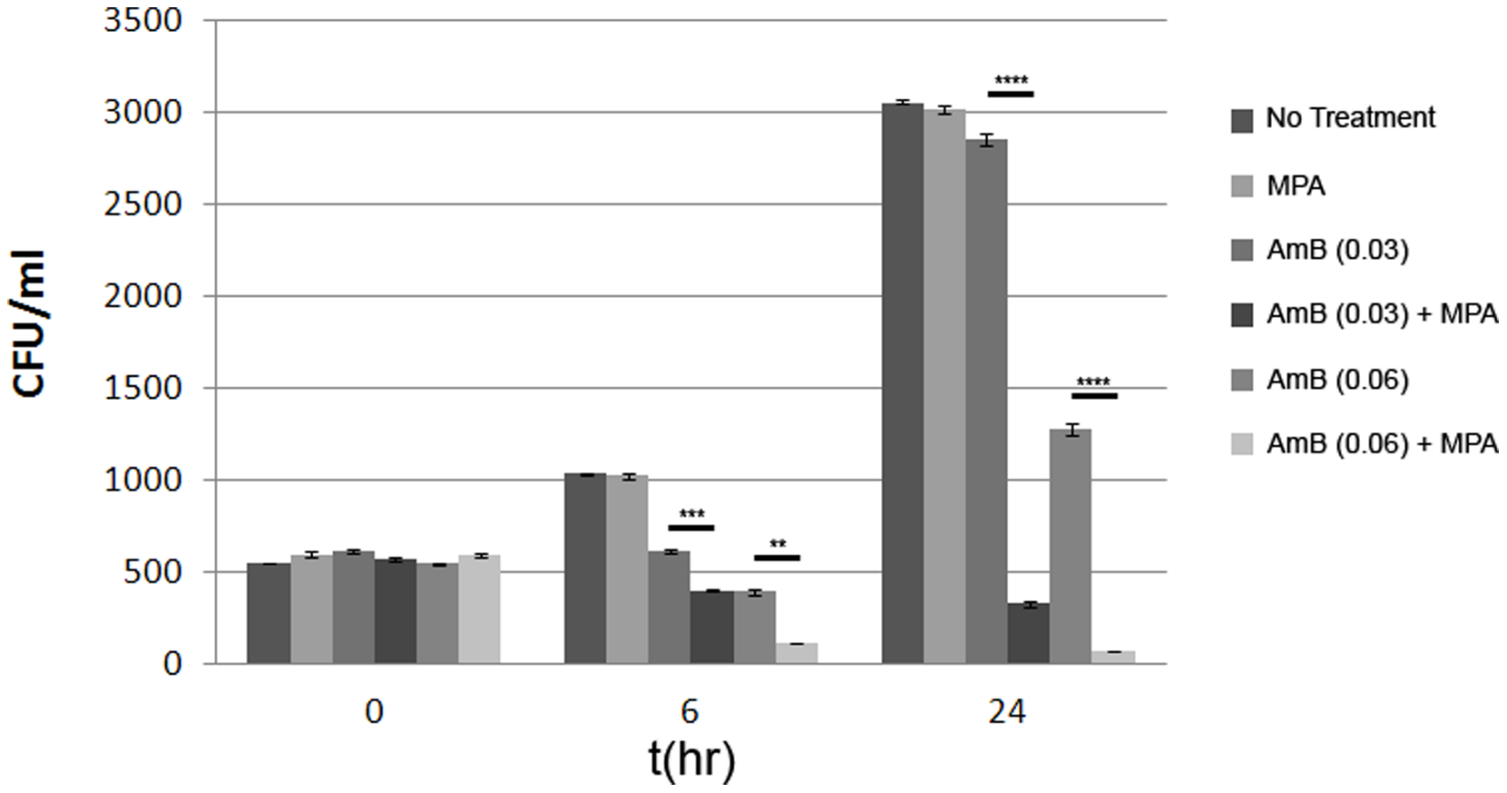
MPA potentiates the killing of *C. neoformans* by AmB. Time kill assay of *C. neoformans* in the presence of AmB at either 0.03 µg/mL or 0.06 µg/mL of AmB alone or in combination with 30 µg/mL MPA. CFU were enumerated from triplicate plates. The graph represents mean CFU +/− SEM (****p<0.0001, ***p<0.001, **p<0.01, *p<0.05).

From our previous data with E-test and checkerboard assays, we hypothesized that a combination of AmB and MPA would result in more effective and rapid cell death. To investigate the effects of combinations of graded concentrations of AmB (0.03 µg/mL, 0.06 µg/mL) and a fixed concentration of MPA (30 µg/mL) on the survival of *C. neoformans*, we performed time kill assays on the wild type strain following 6 and 24 hours of drug exposure. As demonstrated in Figure 5, marked reductions in the numbers of CFU were observed when AmB was used in combination with 30 µg/mL MPA by 6 hours and the effect was more pronounced by 24 hours post treatment which further confirmed the synergistic action of AmB and IMPDH inhibitor, MPA. This data, taken with evidence from the studies of an IMPDH deletion mutant, suggest that purine biosynthesis may also be a viable therapeutic target for cryptococcal infections [23].

### C. neoformans ura^−^ Mutants Exhibit Capsule Defects

**Figure 6:**
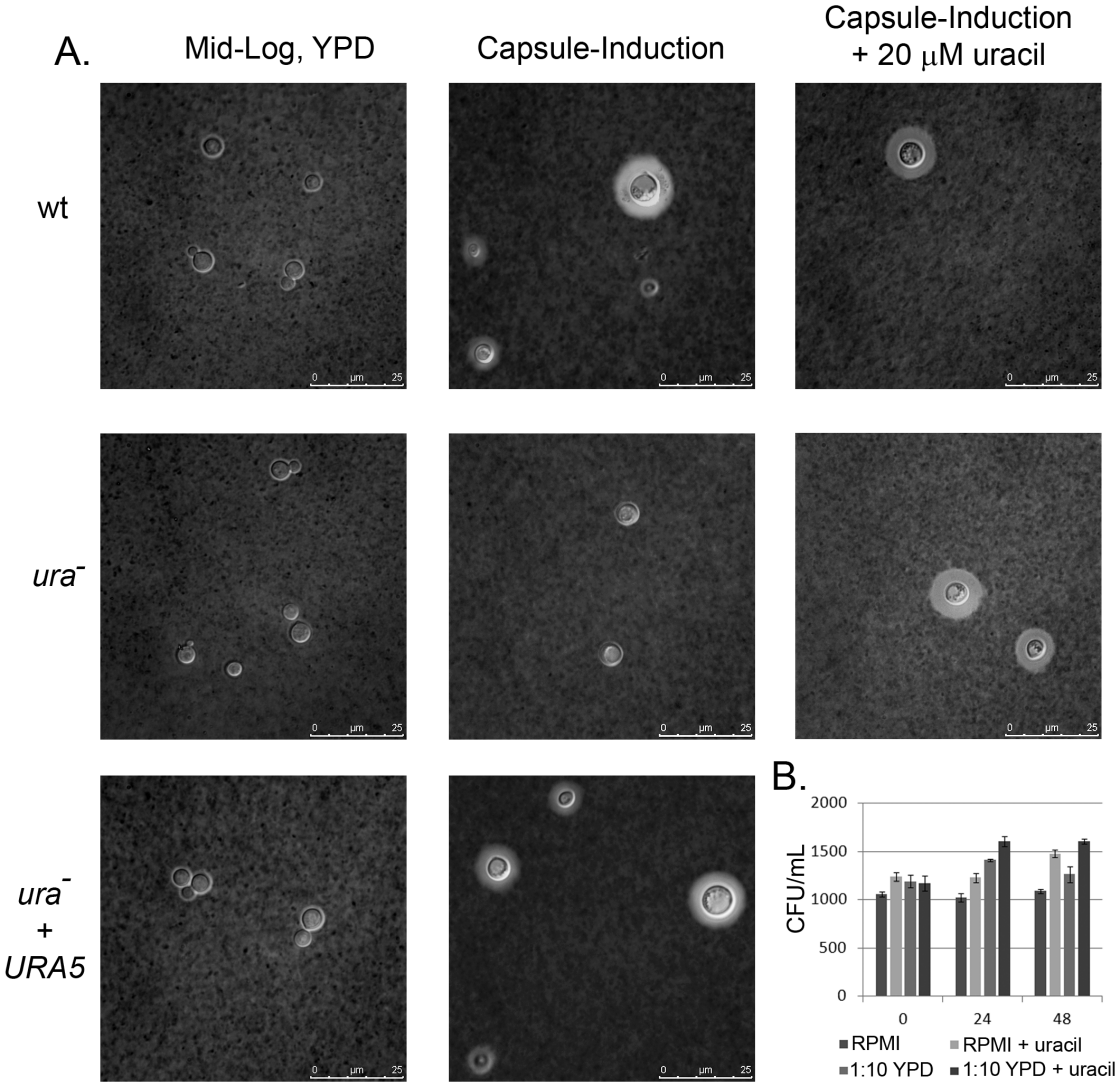
*C. neoformans ura* ^−^ mutants exhibit capsule defects. (A) Representative images of capsule in wt, *C. neoformans ura* ^−^ mutant and *ura* ^−^ complement strains post induction in 1X RPMI at 37°C for 72 hours. (n = 100, represents number of cells measured) (B) Graph representing CFU +/− SEM of *C. neoformans ura* ^−^ mutant in capsule inducing conditions to demonstrate the viability of cells in 1X RPMI or 1∶10 diluted YPD.

We saw from Figure 1-B that uracil or uridine supplementation was insufficient to compensate the enhanced effect of AmB in *C. neoformans*. Keeping in mind the relation between capsule structure and composition with the pathogenic potential of the organism, we reasoned earlier that capsule biosynthesis places an additional draw on nucleotide pools. To address this, we performed capsule detection assay with wild type, *ura* ^−^ mutant and complement strain in the presence and absence of 20µM uracil and uridine. Growth in YPD broth served as a negative control where capsule was not induced in either of the strains. After induction, large capsules (mean radius = 2.76 µm +/−0.09) were present in wild type, which increased in size after addition of uracil (mean radius = 3.03 µm +/−0.08) and uridine (mean radius = 3.33 µm +/−0.1) whereas *ura* ^−^ mutant exhibited marked defect for capsule production (mean radius = 0.369 µm +/−0.07, *p<0.0001* compared to wild type). This defect was recovered by the addition of 20 µM uracil (mean radius = 3.33 µm +/−0.14) and 20 µM uridine (mean radius = 3.33 µm +/−0.09) which suggested that exogenous nucleotides were required for capsule synthesis. Post induction, complement strains developed capsules (mean radius = 3.1026 µm +/−0.09) with comparable size as that of wild type as expected. Figure 6 depicts representative images from these experiments.

To confirm that the absence of capsule in *ura* ^−^ mutant was due to a synthesis defect and not due to cell death in capsule inducing media (1X RPMI or 1∶10 diluted YPD), we performed a survival assay where we took standard aliquots of cells from the capsule inducing media after 24 and 48 hours of incubation and plated them on YPD agar. Results showed that *ura* ^−^ mutant cells were viable in the capsule inducing media thereby attributing its acapsular phenotype to a synthesis defect (Figure 6-B).

### Nucleotide Synthesis Inhibition Renders *C. albicans* More Sensitive to AmB

**Figure 7:**
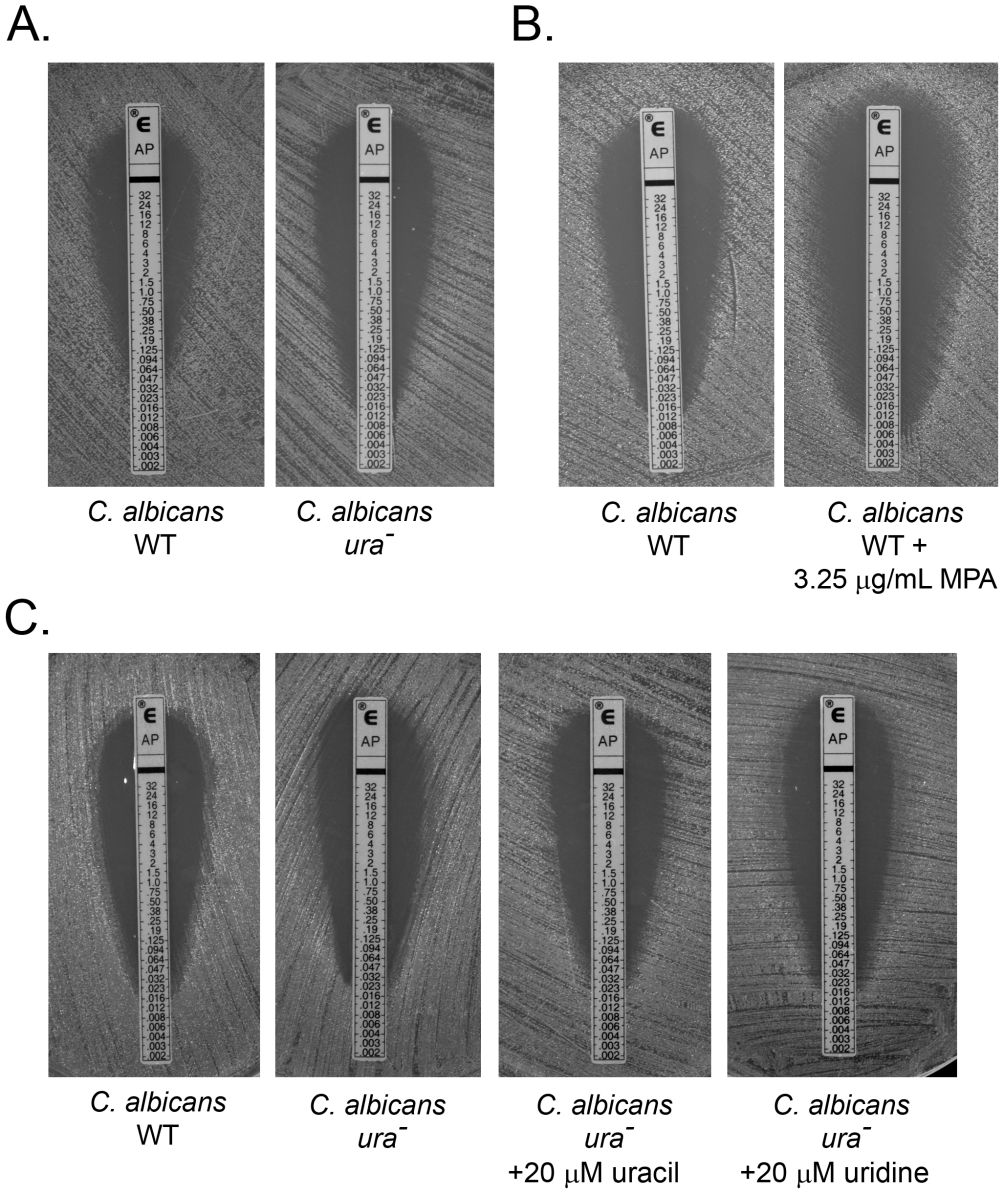
Perturbations in nucleotide biosynthesis sensitize *Candida albicans* to AmB. E-Test analysis for AmB susceptibility of (A) *C. albicans* wt and *ura* ^−^ mutant on AM3 medium (B) *C. albicans* wt on AM3 medium alone or supplemented with 3.25 µg/mL MPA (C) *C. albicans* wt and *ura* ^−^ mutant supplemented with 20 µM uracil or 20 µM uridine.

Once the increased efficacy of AmB was demonstrated in conditions of impaired nucleotide synthesis in *C. neoformans*, we wanted to investigate if this enhanced antifungal effect was consistent in other pathogenic fungi. C. *albicans* is the causative agent of oral thrush, vulvo-vaginal candidiasis and nosocomial disseminated candidiasis. To evaluate the effect of AmB when pyrimidine synthesis was inhibited in *C. albicans*, we performed E-test wild type strain SC5314 and a *ura* ^−^ derivative strain CAI-4. As demonstrated in Figure 7-A, the sensitivity of a *ura* ^−^ strain of *C. albicans* was greater than that of the parental strain with a nearly four-fold reduction in the AmB MIC. This was similar to that seen in *C. neoformans* earlier. To assess whether inhibition of purine biosynthesis altered the efficacy of AmB to *C. albicans*, we performed E-test with wild type *C. albicans* in the presence of 3.25 µg/mL MPA. As demonstrated in Figure 7-B, inhibition of GMP biosynthesis by MPA also increased sensitivity to AmB, reducing the MIC by nearly three-fold. The addition of exogenous 20 µM uracil and 20 µM uridine both restored the wild type MIC levels in the *C. albicans* ura^−^ mutant (Figure 7-C) which demonstrates that this supplementation is enough to compensate for the effect of AmB sensitivity which is in contrast to *C. neoformans* as we saw in Figure1-B. This suggests that fundamental differences in nucleotide pools or utilization of those pools exist between these two pathogens.

**Figure 8:**
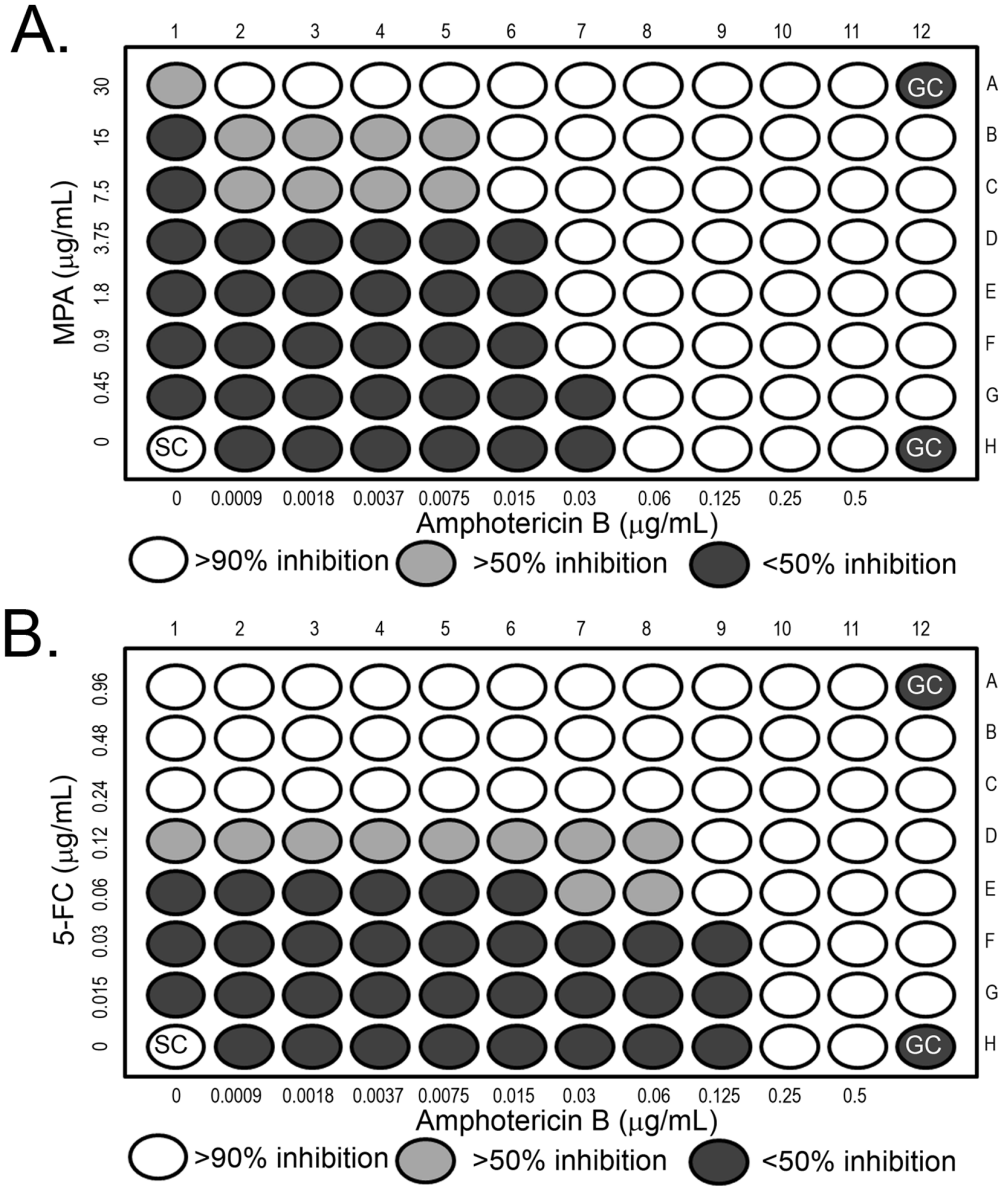
AmB and MPA or 5-FC exhibit positive interactions against *Candida albicans*. (A) Checkerboard analysis of drug interaction between AmB and MPA against *C. albicans.* Assay range of concentrations for AmB was 0.0009 µg/mL to 0.5 µg/mL and MPA was 0.45 µg/mL to 30 µg/mL. (B) Checkerboard analysis of drug interaction between AmB and 5-FC against *C. albicans*. The range of drug concentration used for AmB was the same as in previous experiments, for 5-FC the range was 0.015 µg/mL to 0.96 µg/mL.

We next wanted to investigate the effects of purine and pyrimidine nucleotide inhibitors in combination with AmB. We performed checkerboard assays to study the drug interactions between AmB with MPA and 5-FC against *C. albicans*. In Figure 8-A, combination of AmB and MPA resulted in a decrease in the mean MIC of AmB, from 0.06 µg/mL to 0.015 µg/mL, and MPA, from 30 µg/mL to 7.5 µg/mL. The FIC index from three independent experiments were 0.50, 0.50 and 0.75 which indicated synergy (≤0.5) in the first two cases and additive interaction (0.5 to 1.0) in the third replicate (Table 2). Similarly, in Figure 8-B, interaction between AmB and 5-FC showed a decrease in the MIC of AmB from 0.25 µg/mL to 0.125 µg/mL, and in the MIC of 5-FC from 0.12 µg/mL to 0.06 µg/mL. The FIC index from three independent experiments in this case was reported to be 1.0, 1.0 and 0.75 which indicated additive interactions between the two drugs (Table 3). Our results are comparable to earlier *in vivo* and clinical studies where synergistic or additive interactions have been reported between AmB and 5-FC in *C. albicans* [18], [35], [36]. Taken together, these data demonstrated the increased susceptibility of *C. albicans* to the action of AmB when nucleotide synthesis was inhibited either by the use of a pharmacologic agent or by the loss of a gene in a functional biosynthesis pathway. In addition, conservation of these interactions between the two predominant fungal pathogens suggests that pharmacologic targeting of these pathways may result in similar efficacies, and therefore, a broad spectrum antifungal drug.

**Table 2:** Drug interaction between AmB and MPA in *C. albicans.*

Drugs AmB and MPA (µg/mL)	Replicate 1	Replicate 2	Replicate 3
**AmB alone (MIC_A_)**	0.06	0.06	0.012
**AmB in combination (MIC_A’_)**	0.015	0.015	0.03
**MPA alone (MIC_B_)**	30	30	30
**MPA in combination (MIC_B’_)**	7.5	7.5	15
**FIC index = MIC_A’_/MIC_A_+MIC_B’_/MIC_B_**	0.5	0.5	0.75
**Interaction**	Synergy (≤0.5)	Synergy (≤0.5)	Additive(0.5–1.0)

AmB: Amphotericin B, MPA: Mycophenolic acid.

MICs of AmB and MPA alone and in combination against *C. albicans* in 3 separate experiments and calculated FIC values.

**Table 3:** Drug interaction between AmB and 5-FC in *C. albicans.*

Drugs AmB and 5-FC(µg/mL)	Replicate 1	Replicate 2	Replicate 3
**AmB alone (MIC_A_)**	0.125	0.06	0.012
**AmB in combination (MIC_A’_)**	0.25	0.015	0.03
**5-FC alone (MIC_B_)**	0.12	30	30
**5-FC in combination (MIC_B’_)**	0.06	7.5	15
**FIC index = MIC_A’_/MIC_A_+MIC_B’_/MIC_B_**	1.0	0.5	0.75
**Interaction**	Additive(0.5–1.0)	Additive(0.5–1.0)	Additive(0.5–1.0)

AmB: Amphotericin B, 5-FC: 5-Flourocytosine.

MICs of AmB and 5-FC alone and in combination against *C. albicans* in 3 separate experiments and calculated FIC values.

### Nucleotide Synthesis Inhibition has No Effect on the Susceptibility of *A. fumigatus* to AmB

**Figure 9:**
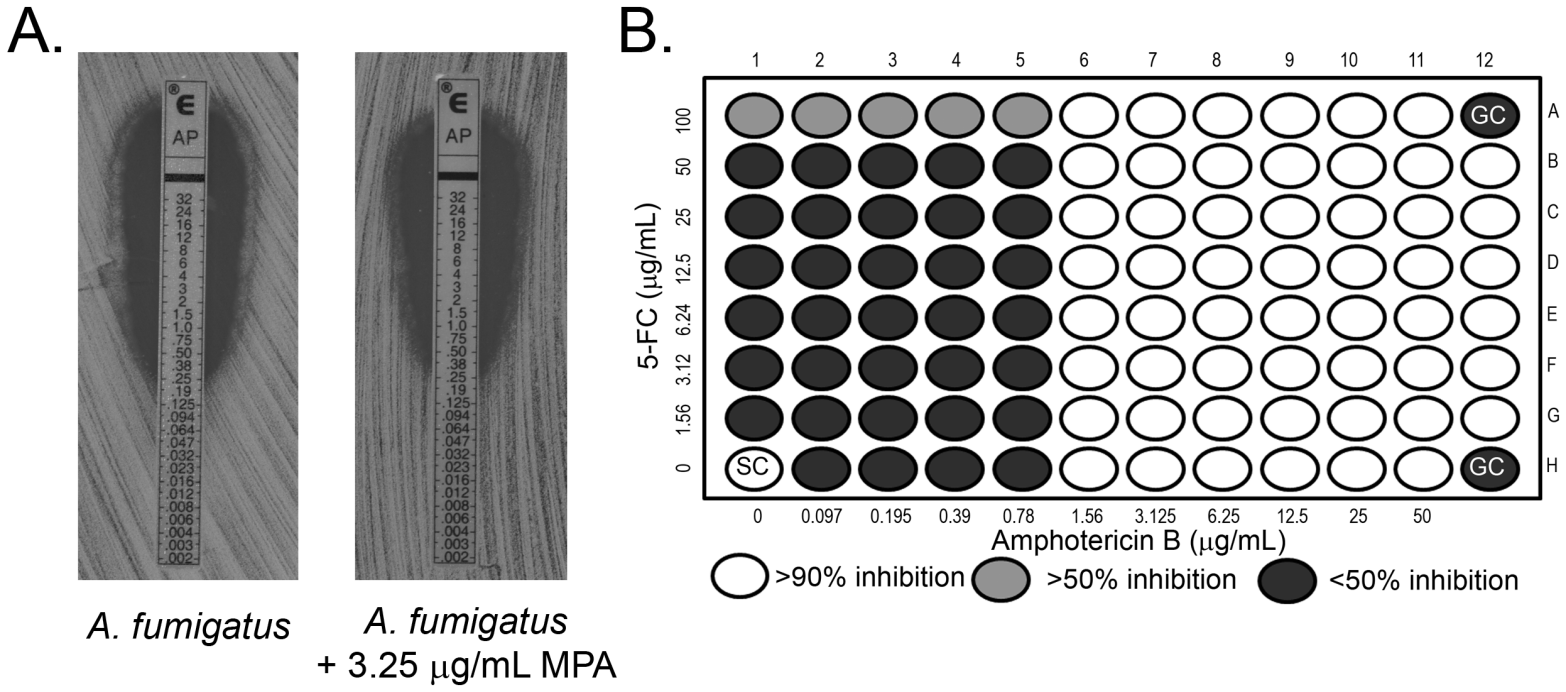
Perturbations in nucleotide metabolism do not alter the AmB susceptibility of *Aspergillus fumigatus*. E-Test analysis of *A. fumigatus* wt on AM3 medium alone or supplemented with 3.25 µg/mL MPA. (B) Checkerboard analysis to assess drug interaction between AmB and 5-FC against *A. fumigatus* using the following drug ranges; AmB 0.097 µg/mL to 50 µg/mL and 5-FC from 1.56 µg/mL to 100 µg/mL.

*A. fumigatus* is another well-known fungal pathogen against which AmB is used, and is the predominant mold pathogen of humans [37]–[39]. To investigate whether the efficacy of the AmB was altered when nucleotide synthesis was inhibited, we performed E-test with wild type strain of *A. fumigatus* using a combination of AmB and MPA. In Figure 9-A, there was no change in MIC during combination treatment as compared to AmB alone and the MIC was reported as 1.25 µg/mL under both conditions. Earlier in vitro studies with *A. fumigatus* strains from clinical isolates showed no significant interaction between AmB and 5-FC with the MIC levels of AmB remaining unchanged, showing antagonism or additive effect with the latter being inconsistent in most cases [40]–[42]. Because the previous studies were performed in YNB-Dex or Eagle minimal essential medium, we performed these same checkerboard assays using in RPMI 1640–2% Dex for consistency with our studies of *C. neoformans* and *C. albicans* presented above. In Figure 9-B the MIC of AmB was consistent at 1.56 µg/mL whereas there was no significant killing (>50%) observed by 5-FC. The FIC indices of three independent experiments were 2, 2 and 1.5 which indicated indifferent interaction (1–4) (Table 4). These results suggest that *A*. *fumigatu*s is a more potent pathogen requiring higher doses (higher MIC) of drug for its inhibition which does not change, even in the presence of drugs that perturb nucleotide biosynthesis.

**Table 4:** Drug interaction between AmB and 5-FC against *A. fumigatus.*

Drug (µg/mL)	Replicate 1	Replicate 2	Replicate 3
**AmB alone (MIC_A_)**	1.56	1.56	3.125
**AmB in combination (MIC_A’_)**	1.56	1.56	1.56
**5-FC alone (MIC_B_)**	100	100	100
**5-FC in combination (MIC_B’_)**	100	100	100
**FIC index = MIC_A’_/MIC_A_+MIC_B’_/MIC_B_**	2.0	2.0	1.5
**Interaction**	Indifference (1–4)	Indifference (1–4)	Indifference (1–4)

AmB: Amphotericin B, 5-FC: 5-Flourocytosine.

MICs of AmB and 5-FC alone and in combination against *A. fumigatus* in 3 separate experiments and calculated FIC values.

We see a distinct difference in the AMB susceptibility pattern in the three fungal species - *C. neoformans*, *C. albicans* and *A. fumigatus* and we attribute it to the basic differences in their structure and physiology. Since *C. neoformans* is an encapsulated pathogen, it requires uridine and uracil nucleotides for its capsule synthesis and so exogenous supplementation may not be sufficient for the *ura* ^−^ mutant to recover from AmB sensitivity. The lack of capsule in *C. albicans* may enable it to utilize exogenous uracil and uridine for its growth, compensating for the AmB effect. *A. fumigatus* on the other hand is a saprophyte which has numerous biochemical processes to utilize complex molecules for their growth and metabolism. Possibly, disrupting IMPDH activity by the use of MPA results in some compensatory process by which it can form or recycle guanosine nucleotides from other sources for its growth. Alternatively, MPA may act specifically on yeast IMPDH and the same enzyme in *A. fumigatus* may not affected by the drug. Nucleotide metabolism has not been explored extensively in *A. fumigatus* and these interesting results can lead to further studies in this field. The observation that 5-FC had no effect on AmB susceptibility suggests that a more robust salvage pathway may be present in this environmental saprophyte.

Invasive fungal infections are increasing both in the US and abroad, demonstrating a clear need for novel antifungal therapies [43]–[46]. The therapeutic potential of AmB is limited by its toxicity, despite its potent antifungal activity. In this study, we report that pharmacologic targeting of nucleotide biosynthesis pathways can lower the effective concentration of AmB *in vitro*, suggesting that novel adjunctive therapies may be identified that might allow AmB to be used at doses that are safe as well as effective. Future work will identify specific targets in these pathways for development of new antifungal therapies to either augment or replace existing therapeutic agents.
